# Draft genomes and descriptions of *Urmitella timonensis* gen. nov., sp. nov. and *Marasmitruncus massiliensis* gen. nov., sp. nov*.,* isolated from severely malnourished African children using culturomics

**DOI:** 10.1007/s10482-022-01777-x

**Published:** 2022-09-23

**Authors:** Sara Bellali, Gabriel Haddad, Thi-Phuong-Thao Pham, Rim Iwaza, Ahmad Ibrahim, Nicholas Armstrong, Amael Fadlane, Carine Couderc, Aldiouma Diallo, Cheikh Sokhna, Matthieu Million, Didier Raoult, Maryam Tidjani Alou

**Affiliations:** 1 Aix Marseille Université, MEPHI, URMITE, UM63, CNRS 7278, IRD 198, INSERM 1095, Marseille, France; 2 IHU Méditerranée Infection, 19-21 Boulevard Jean Moulin, 13385 Marseille, France; 3 Campus Commun UCAD-IRD of Hann, Dakar, Senegal; 4 Aix Marseille Univ, IRD, AP-HM, SSA, VITROME, Marseille, France

**Keywords:** *Marasmitruncus massiliensis*, *Urmitella timonensis*, Culturomics, Taxono-genomics, Human gut microbiota, New taxa

## Abstract

Two strains, designated as Marseille-P2918^T^ and Marseille-P3646^T^, were isolated from a 14-week-old Senegalese girl using culturomics: *Urmitella timonensis* strain Marseille-P2918^T^ (= CSUR P2918, = DSM 103634) and *Marasmitruncus massiliensis* strain Marseille-P3646^T^ (= CSUR P3646, = CCUG72353). Both strains were rod-shaped, anaerobic, spore forming motile bacteria. The 16S rRNA gene sequences of strains Marseille-P2918^T^ (LT598554) and Marseille-P3646^T^ (LT725660) shared 93.25% and 94.34% identity with *Tissierella praeacuta* ATCC 25539^T^ and *Anaerotruncus colihominis* CIP 107754^T^, their respective phylogenetically closest species with standing in nomenclature. Therefore, strain Marseille-P2918^T^ is classified within the family *Tissierellaceae* and order *Tissierellales* whereas strain Marseille-P3646^T^ is classified within the family *Oscillospiraceae* and order *Eubacteriales*. The genome of strain Marseille-P2918^T^ had a size of 2.13 Mb with a GC content of 50.52% and includes six scaffolds and six contigs, and that of strain Marseille-P3646^T^ was 3.76 Mbp long consisting of five contigs with a 50.04% GC content. The genomes of both strains presented a high percentage of genes encoding enzymes involved in genetic information and processing, suggesting a high growth rate and adaptability. These new taxa are extensively described and characterised in this paper, using the concept of taxono-genomic description.

**Supplementary Information:**

The online version contains supplementary material available at 10.1007/s10482-022-01777-x.

## Introduction

Severe acute malnutrition (SAM) is a life-threatening condition requiring urgent treatment. It is responsible, directly or indirectly, for around 45% of the child mortality rate worldwide (World Health Organization 2021). Clinically, there are three forms of SAM: marasmus, characterised by a severe loss of subcutaneous fat and extreme wasting, kwashiorkor, characterised by the presence of an oedema, and marasmic kwashiorkor which combines extreme wasting and oedema. To improve understanding the pathogenesis of SAM and develop more effective therapeutic strategies, various studies have been conducted (Mata et al. 1972; Gupta et al. 2011; Monira et al. 2011; Smith et al. 2013; Ghosh et al. 2014; Subramanian et al. 2014; Million et al. 2016). Smythe (1958) was among the first to link kwashiorkor to an alteration of the intestinal bacterial microbiota in the 1950s. A study by Tidjani Alou et al*.* (2017) further demonstrated the link between kwashiorkor and a dysbiosis of the gut microbiota, highlighting a significant decrease in anaerobic bacteria and revealing a hitherto unknown diversity using metagenomics and culturomics.

A similar study conducted in 2016 aimed at characterising the gut microbiota of children suffering from marasmus. This study not only characterised more precisely the dysbiosis associated with children suffering from marasmus, but also led to the isolation of 20 new species. Two previously unknown bacterial strains, Marseille-P2918^T^ and Marseille-P3646^T^, were thus isolated as a part of this study. These two strains were classified within the families *Tissierellaceae* and *Oscillospiraceae*, respectively. The family *Tissierellaceae* was created in 2014 (Alauzet et al. 2014), validated in 2020 (Wu et al. 2020) and currently consists of eight validly published genera (https://lpsn.dsmz.de/family/tissierellaceae, last accessed August 23rd). The family *Oscillospiraceae* was validated in 1980 (Sneath et al. 1980) and emended to the “family *Ruminococcaceae*” in 2010 (Euzeby 2010). There are currently 56 genera included in this family (https://lpsn.dsmz.de/family/oscillospiraceae, last accessed August 23rd), according to the List of Prokaryotic names with Standing in Nomenclature (LPSN (Parte et al. 2020)). Members of these families are described mostly as anaerobic and can be either Gram-stain positive or negative. The bacterial cells of some species can be either nonmotile or motile. These two new genera, classified within the families *Tissierellaceae* and *Oscillospiraceae*, are here described using the taxono-genomics concept (Fournier et al. 2015) which consists in the classification and characterisation of new bacterial strains based on phylogenetic, phylogenomic and phenotypic characteristics., thus providing insight into their fitness for the gut of a malnourished child.

## Material and methods

### Sample collection

Stool samples were collected from a 14-week-old Senegalese girl diagnosed with marasmus based on anthropometric criteria. Written consent from the parents of the patient and the agreement of the local ethics committee under protocol number SEN16/45, as well as that of the Institut Fédératif de Recherche 48 (IFR48) were obtained (agreement number 09–022, Marseille, France). The stool sample was stored at − 80 °C after collection and sent to the La Timone hospital (Marseille, France).

### Strain identification and phylogenetic analysis

The microbial diversity of the sample was assessed using the 18 standard culture conditions of culturomics as previously described by Lagier et al. (2015). Enriched blood culture bottles were monitored every three days for a month following inoculation by seeding the culture on 5% sheep blood enriched Columbia (COS) agar (bioMérieux, Craponne, France). All colonies were identified using MALDI-TOF MS (Seng et al. 2009). The obtained spectra were compared with the Bruker database and that of the La Timone hospital. Colonies were labelled as correctly identified at the species level with a score ≥ 2, at the genus level with a score between 1.7 and 2, and as unidentified with a score < 1.7. Unidentified strains using MALDI-TOF MS underwent Sanger sequencing of the 16S rRNA gene using the fD1 and rP2 primer pair (Drancourt et al. 2000). The obtained sequences were assembled and corrected using the CodonCode Aligner software (http://www.codoncode.com) and then compared with the sequences available in the GenBank nucleotide database using BLASTn (http://blast.ncbi.nlm.nih.gov.gate1.inist.fr/Blast.cgi). A similarity threshold under 98.7% was used to define a new species whereas a threshold under 95% was used to define a new genus (Auch et al. 2010). For the phylogenetic analysis, a ClustalW alignment on the collected 16S rRNA sequences was performed using MEGA7. These alignments allowed the construction of a phylogenetic tree which was computed using the Neighbor Joining method as well as the Maximum Likelihood method with 1000 bootstrap replicates, based on the Tamura-Nei model (1993). Codon positions included were first, second, third, and noncoding. The analysis involved 19 and 17 nucleotide sequences and a total of 1495 and 1435 nucleotides in the final datasets of Marseille-P2918^T^ and Marseille-P3646^T^ respectively. All positions containing gaps and missing data were eliminated. The analyses were conducted within the MEGA7 software (Kumar et al. 2016).

### Phenotypic features

As previously described, phenotypic characteristics such as Gram staining, motility, and sporulation were assessed (Lagier et al. 2016). The optimal growth condition on COS agar was also determined by testing seven growth temperatures (20, 25, 28, 30, 37, 45 and 56 °C) under an aerobic atmosphere with or without 5% CO_2_, as well as under anaerobic and microaerophilic atmospheres generated using anaeroGEN (Beckton Dickinson, Arcueil, FRANCE) and campyGEN (Beckton Dickinson) generators respectively.

Additionally, a fresh colony was observed through a Leica DM 1000 photonic microscope (Leica Microsystems, Wetzlar, Germany) at a 40 × magnification to assess the motility of the bacteria. Morphological features of the strains were further described using electron microscopy. Pure cultures were cyto-centrifuged on glass slides and sputtered with a 5 µm thick platinum layer using ion sputter MC1000 (Hitachi, Japan). Slides were imaged on a SU5000 scanning electron microscope (SEM) (Hitachi, Japan). Micrographs were acquired at magnifications ranging from × 10,000 to × 50,000 with a 10 kV voltage and a spot intensity of 30 using the backscatter electron detector in high vacuum mode. Biochemical analysis of strains Marseille-P2918^T^ and Marseille-P3646^T^ was carried out using API 50CH, API 20A, API ZYM strips (bioMérieux) according to the manufacturer’s instructions. The presence of catalase (bioMérieux) and oxidase (Becton Dickinson, Franklin Lakes, NJ, USA) activities was also assessed.

Fatty acid methyl esters were prepared (Sasser 1990) prior to GC/MS analyses being carried out as previously described (Dione et al. 2016). Briefly, fatty acid methyl esters were separated using an Elite 5-MS column and monitored by mass spectrometry (Clarus 500—SQ 8 S, Perkin Elmer, Courtaboeuf, France). A spectral database search was performed using MS Search 2.0 operated with the Standard Reference Database 1A (NIST, Gaithersburg, USA) and the FAMEs mass spectral database (Wiley, Chichester, UK).

Antibiotic susceptibility was evaluated using the disc diffusion method and E-test strips (bioMérieux) according to the EUCAST recommendations (www.eucast.org). E-test strips were used to determine the minimal inhibitory concentration (MIC) of the following antibiotics: benzylpenicillin, oxacillin, ceftazidime, tobramycin, amikacin, amoxicillin, ceftriaxone, imipenem, vancomycin, doxycycline, clindamycin, trimethoprim/sulfamethoxazole, ciprofloxacin, rifampicin, linezolid and colistin.

### Genome description and comparison

Genomic DNA (gDNA) of the two described strains was extracted on the EZ1 biorobot (Qiagen) with an EZ1 DNA tissue kit after a two hour-lysozyme incubation at 37 °C. gDNA was then quantified using a Qubit assay with the high sensitivity kit (Life technologies, Carlsbad, CA, USA). Sequencing of gDNA was carried out using the MiSeq Technology (Illumina Inc, San Diego, CA, USA) with the mate pair strategy. The gDNA was then barcoded to be mixed with 11 other projects with the Nextera Mate Pair sample prep kit (Illumina). The Nextera Mate Pair Illumina guide was used to prepare the mate pair library. The gDNA sample was simultaneously fragmented and tagged with a mate pair junction adapter. The pattern of the fragmentation was validated on an Agilent 2100 BioAnalyzer (Agilent Technologies Inc, Santa Clara, CA, USA) with a DNA 7500 LabChip. The library profile was visualised on a High Sensitivity Bioanalyzer LabChip (Agilent Technologies Inc, Santa Clara, CA, USA) and the final concentration library was measured.

The genomes of strains Marseille-P2918^T^ and Marseille-P3646^T^ and those of their closest phylogenetic relatives were annotated using Prokka (Seemann 2014). A map of the circular genome was also built using CGview to display the genomic features of these new taxa (Petkau et al. 2010). For genomic comparison, the OAT software (Lee et al. 2016) was used to build an OrthoANI heatmap in order to estimate the average nucleotide identity at the genomic level between strain Marseille-P2918^T^, Marseille-P3646^T^ and closely related species. Similarly, OrthoAAI (Average Amino-acid Identity) was determined for each strain using AAI-profiler online tool (Medlar et al. 2018). Moreover, genomic similarity was further determined using digital DNA-DNA hybridization (dDDH) which was calculated using Type (Strain) Genome Server (TYGS) (https://tygs.dsmz.de/) (Auch et al. 2010; Meier-Kolthoff et al. 2013). For the proteomics analyses, we performed a BLASTp analysis for our strains and closely related species genomes against the clusters of orthologous groups (COG) database with a minimum identity of 30%, a minimum coverage of 70%, and a maximum E-value of 1e^−03^. All sequences shorter than 80 amino acids in size were removed. In addition, for the metabolic pathway determination, we used BLASTp Koala against KEGG prokaryotic database (Kanehisa et al. 2016).

Antimicrobial resistance screening was achieved through the analysis of nucleotide sequences of each genome using ABRicate against ResFinder and PlasmidFinder databases (Zankari et al. 2012; Carattoli et al. 2014). Moreover, we applied the strategy recently published by Khabthani et al. (Khabthani et al. 2021) to look for new antibiotic resistance genes in each described genomes using protein sequences and CDD confirmation (Maatouk et al. 2021).

## Results

### Phenotypic and biochemical characterisation

**Fig. 1:**
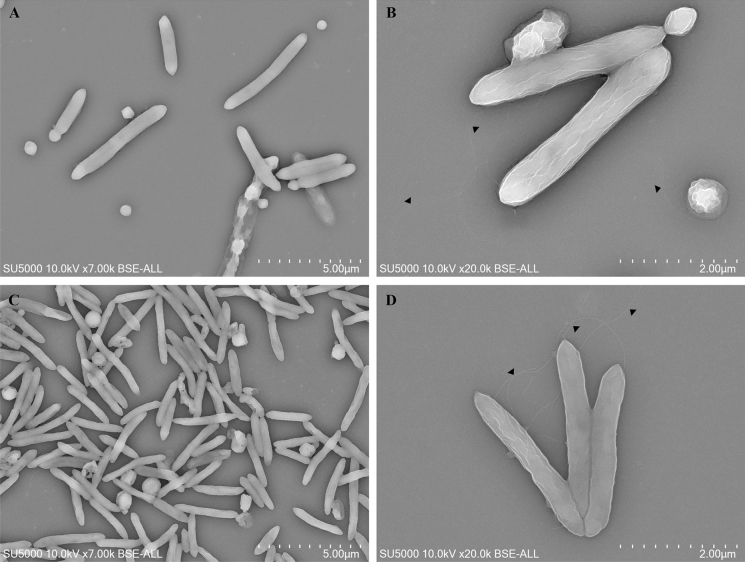
Scanning electron microscopy of *Urmitella timonensis* gen. nov., sp. nov. (**A**, **B**) and *Marasmitruncus massiliensis* gen. nov., sp. nov. (**C**, **D**). Scale bars and acquisition settings are displayed on the figure

The main phenotypic and biochemical features obtained experimentally of our strains were compared with features of the closest species with a valid publication, *Tissierella praeacuta* strain ATCC 25539^T^ (Collins and Shah 1986) for strain Marseille-P2918^T^, and *Anaerotruncus colihominis* CIP 107754^ T^ (Lawson et al. 2004) for strain Marseille P3646^T^, described in Table 1. Strains Marseille-P3646^T^ and Marseille-P2918^T^ were both Gram-stain negative, spore forming, motile bacteria. The growth of strain Marseille-P3646^T^ occurred under anaerobic and microaerophilic atmospheres whereas strain Marseille-P2918^T^ only grew under strictly anaerobic conditions. Growth only occurred at 37 °C within a pH range of 6–8.5 for both strains whereas no growth was observed at 20, 25, 28, 30, 45 and 56 °C. Neither oxidase nor catalase activities were found in both strains. SEM observations revealed that both strains had a similar morphology. They were both rod-shaped and occurred mostly as single cells or in pairs and rarely in chains of more than two cells. Flagella and spores were also visible on the micrographs of both strains (Fig. 1). Cell sizes were 5.33 ± 1.5 µm in length and 0.70 ± 0.09 µm in width for strain Marseille-P2918^T^, and 2.71 ± 0.6 µm in length and 0.42 ± 0.06 µm in width for strain Marseille-P3646^T^.

**Table 1:** Comparison of phenotypic characteristics

Properties	*Urmitella timonensis*	*Tissierella praeacuta*	*Marasmitruncus massiliensis*	*Anaerotruncus colihominis*
Strain	Marseille-P2918^T^	ATCC 25539^ T^	Marseille-P3646^T^	CIP 107754^T^
Sample origin	Human stool	Human stool	Human stool	Human stool
Patient information	Marasmus Senegalese girl	Infant	Marasmus Senegalese girl	71-month-old male child
Isolation conditions	Blood culture + 5% sheep blood Day 7 anaerobic 37 °C	ND	Blood culture + 5% sheep blood Day 7 anaerobic 37 °C	Brucella blood agar, anaerobic 37 °C
Optimal temperature	37 °C	37 °C	37 °C	36–40 °C
Atmosphere	anaerobic	anaerobic	Anaerobic	Anaerobic
pH range	6–8.5	ND	6–8.5	5.5–11
Colony aspect	Circular, smooth, very small and pale grey	Small, circular low convex, greyish, and smooth	Circular, smooth, very small	Grey, entire-edged, irregularly shaped, translucent
Cell shape	Rod-shaped	Circular	Rod	Rod
Cell size (µm)	1.5–4.5	0.6–0.9	0.42–0.5	0.5
	0.5–1.2	2.0–8.0	2.71–3.3	2–5
Gram-stain	Negative	Positive	Negative	Positive
Motility	Yes	Yes	Yes	No
Spore formation	Yes	Yes	Yes	No
Major cellular fatty acid	16:0 (40%)	13-methyltetradecanoic (iso-C15.0)	12-methyl-tetradecanoic acid (15:0 anteiso)	ND
GC content (%)	50.50%	28.00%	50.04%	54%
Production of:				
Catalase	−	ND	−	−
Oxidase	−	ND	−	ND
Urease	−	−		−
Habitat	Human gut	Human gut	Human gut	Human gut

+  = positive, − = negative, ND = no data available

Using an API ZYM strip, both strains exhibited positive reactions for naphthol-AS-BI-phosphohydrolase and acid phosphatase. Additionally, strain Marseille-P2918^T^ exhibited positive reactions for α-glucosidase, leucine arylamidase and alkaline phosphatase as well (Table S1). Using an API 20A strip, positive reactions were obtained for D-glucose for both strains (Table S2). Both strains were also able to metabolize a wide array of carbohydrates (Table S3) as revealed by the API 50CH strip.

The cellular fatty acid analysis revealed that strain Marseille-P2918^T^ consisted mostly of C16-C18 structures: hexadecanoic acid (40%), 9-octadecenoic acid (25%), octadecanoic acid (9%) and 9,12-octadecadienoic acid (9%) whereas the major fatty acids in strain Marseille-P3646^T^ were C15-C16 saturated and branched structures: 12-methyl-tetradecanoic acid (39%), 13-methyltetradecanoic acid (23%) and hexadecanoic acid (15%). Fatty acids are described in Table S4. Additionally, the antibiotic susceptibility of our strains against a selection of molecules was tested and reported in Table S5. Interestingly, MICs > 256 ug/ml were obtained for colistin for both strains as well amikacin for strain Marseille-P2918^T^ and tobramycin for strain Marseille-P3646^T^ suggesting a natural resistance towards these molecules. Conversely, no growth was obtained for both strains for doxycycline and clindamycin as well as ceftazidime, amoxicillin, ceftriaxone and linezolid suggesting a sensibility towards these molecules.

### Strain identification and phylogenetic analysis

**Fig. 2:**
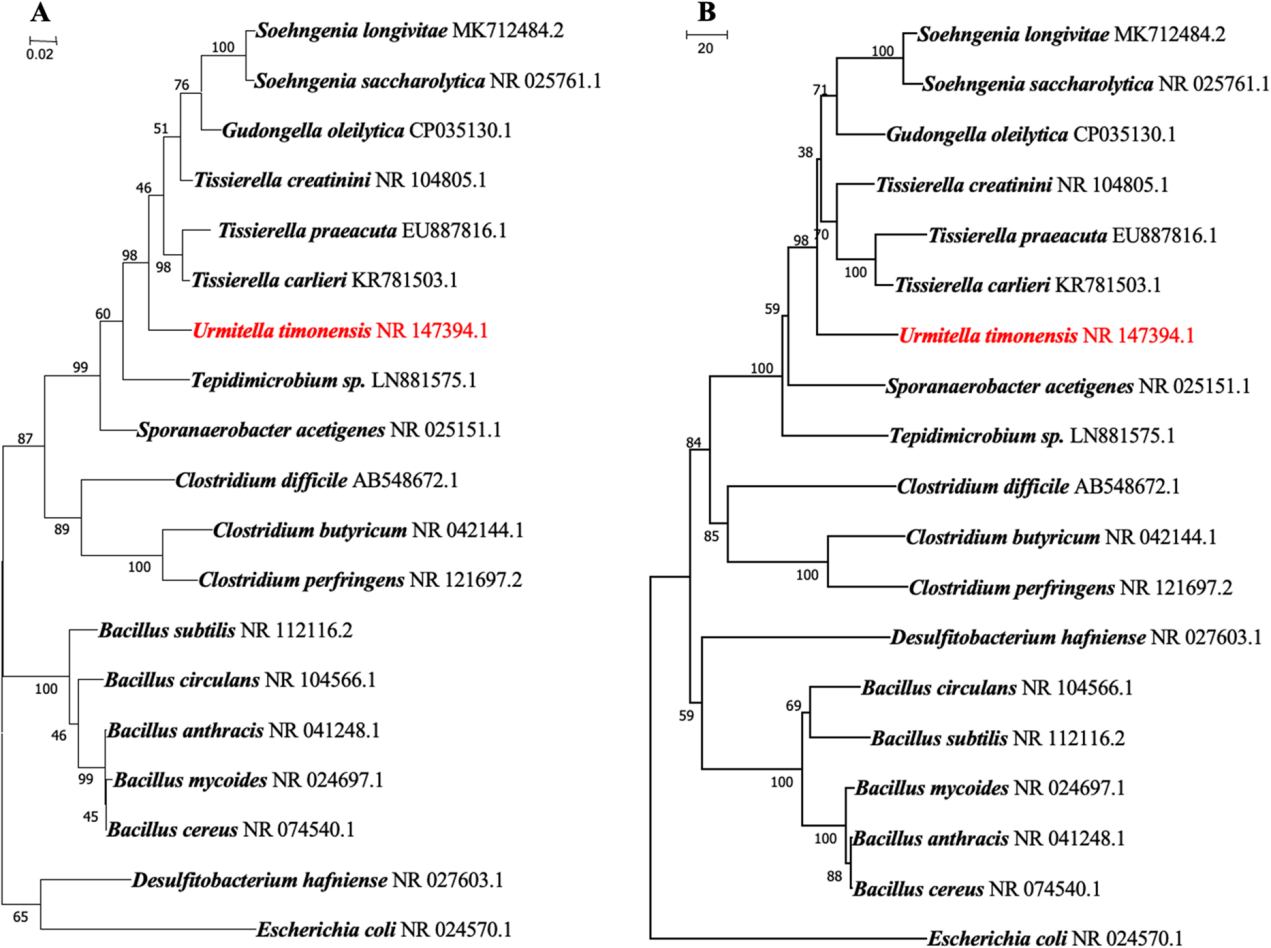
Phylogenetic trees highlighting position of *Urmitella timonensis* strain Marseille-P2918^T^ (in red). Codon positions included were 1st + 2nd + 3rd + Noncoding. All positions containing gaps and missing data were eliminated. There were a total of 1212 positions in the final dataset. Evolutionary analyses were conducted in MEGA7. *Escherichia coli* (NR_024570.1) were used as outgroup. **A** The evolutionary history was inferred by using the Maximum Likelihood method based on the Tamura-Nei model [1]. The tree with the highest log likelihood (− 7626.34) is shown. The tree is drawn to scale, with branch lengths measured in the number of substitutions per site. The analysis involved 20 nucleotide sequences. **B** The tree was built using the Neighbor-Joining method. The optimal tree with the sum of branch length = 966.65234375 is shown. The percentage of replicate trees in which the associated taxa clustered together in the bootstrap test (1000 replicates) are shown next to the branches. The tree is drawn to scale, with branch lengths in the same units as those of the evolutionary distances used to infer the phylogenetic tree. The evolutionary distances were computed using the Tamura-Nei method and are in the units of the number of base substitutions per site. The analysis involved 19 nucleotide sequences.

**Fig. 3:**
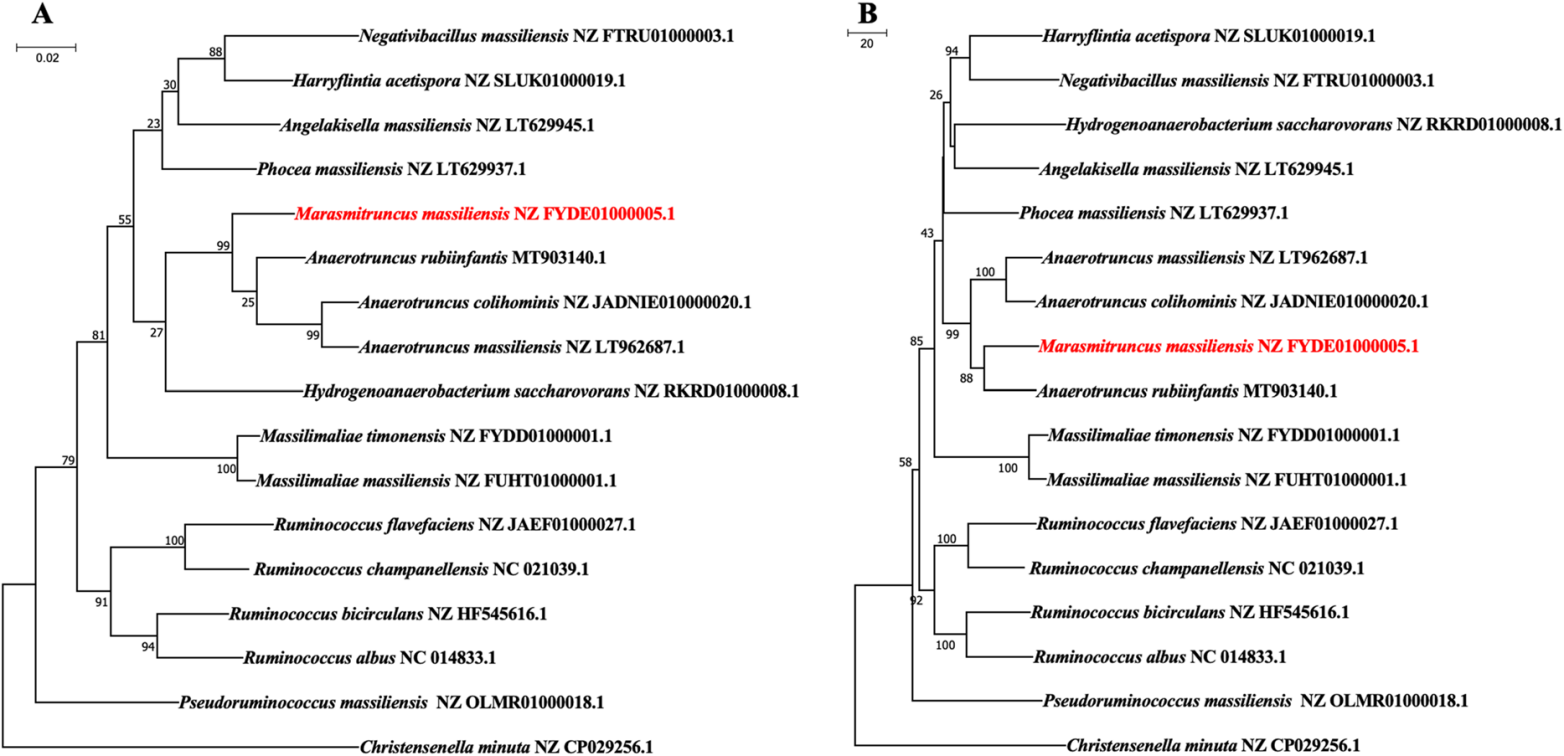
Phylogenetic trees highlighting position of *Marasmitruncus massiliensis* strain Marseille-P3646^T^ (in red). Codon positions included were 1st + 2nd + 3rd + Noncoding. All positions containing gaps and missing data were eliminated. There were a total of 1336 positions in the final dataset. Evolutionary analyses were conducted in MEGA7. *Christensenella minuta* (NZ_CP029256.1) were used as outgroup. **A** The evolutionary history was inferred by using the Maximum Likelihood method based on the Tamura-Nei model [1]. The tree with the highest log likelihood (− 6998.48) is shown. The tree is drawn to scale, with branch lengths measured in the number of substitutions per site. **B** The tree was built using the Neighbor-Joining method. The optimal tree with the sum of branch length = 827.00000000 is shown. The percentage of replicate trees in which the associated taxa clustered together in the bootstrap test (1000 replicates) are shown next to the branches. The tree is drawn to scale, with branch lengths in the same units as those of the evolutionary distances used to infer the phylogenetic tree. The evolutionary distances were computed using the Tamura-Nei method and are in the units of the number of base substitutions per site. The analysis involved 17 nucleotide sequences

The MALDI-TOF MS analysis did not allow identification of our strains of interest. However, the 16S rRNA gene sequence of strain Marseille-P2918^T^ (GenBank accession number LT598554) shared 93.71% identity with that of *Tissierella* *praeacuta* strain NCTC 11,158 (Genbank accession number X80832), the phylogenetically closest species with standing in nomenclature which putatively classifies it as a member of the family *Tissierellaceae* within the phylum *Firmicutes*. Strain Marseille-P2918^T^ exhibits a 16S rRNA sequence divergence over 5% with its phylogenetically closest relative with standing in nomenclature (7). As for strain Marseille-P3646^T^ (Genbank accession number LT725660), its highest sequence similarity, 93.11%, was shared with the 16S rRNA gene of *Anaerotruncus colihominis* strain 14565^ T^ (Genbank accession number AJ315980) which represents a sequence divergence over 5%. The sequences of the 16S rRNA gene of strains Marseille-P2918^T^ and Marseille-P3646^T^ were also compared to those of type species with a validly published name within their respective families and exhibited sequence divergences all over 5% (Table S6). The spectra of the described strains (Figure S1) were incremented to the URMITE database (http://www.mediterranee-infection.com/article.php?laref=256&titre=urms-database). The position of our strains was also assessed by computing phylogenetic trees highlighting their position relative to closely related species (Figs. 2, 3 and S2).

### Genome annotation and comparison

**Fig. 4:**
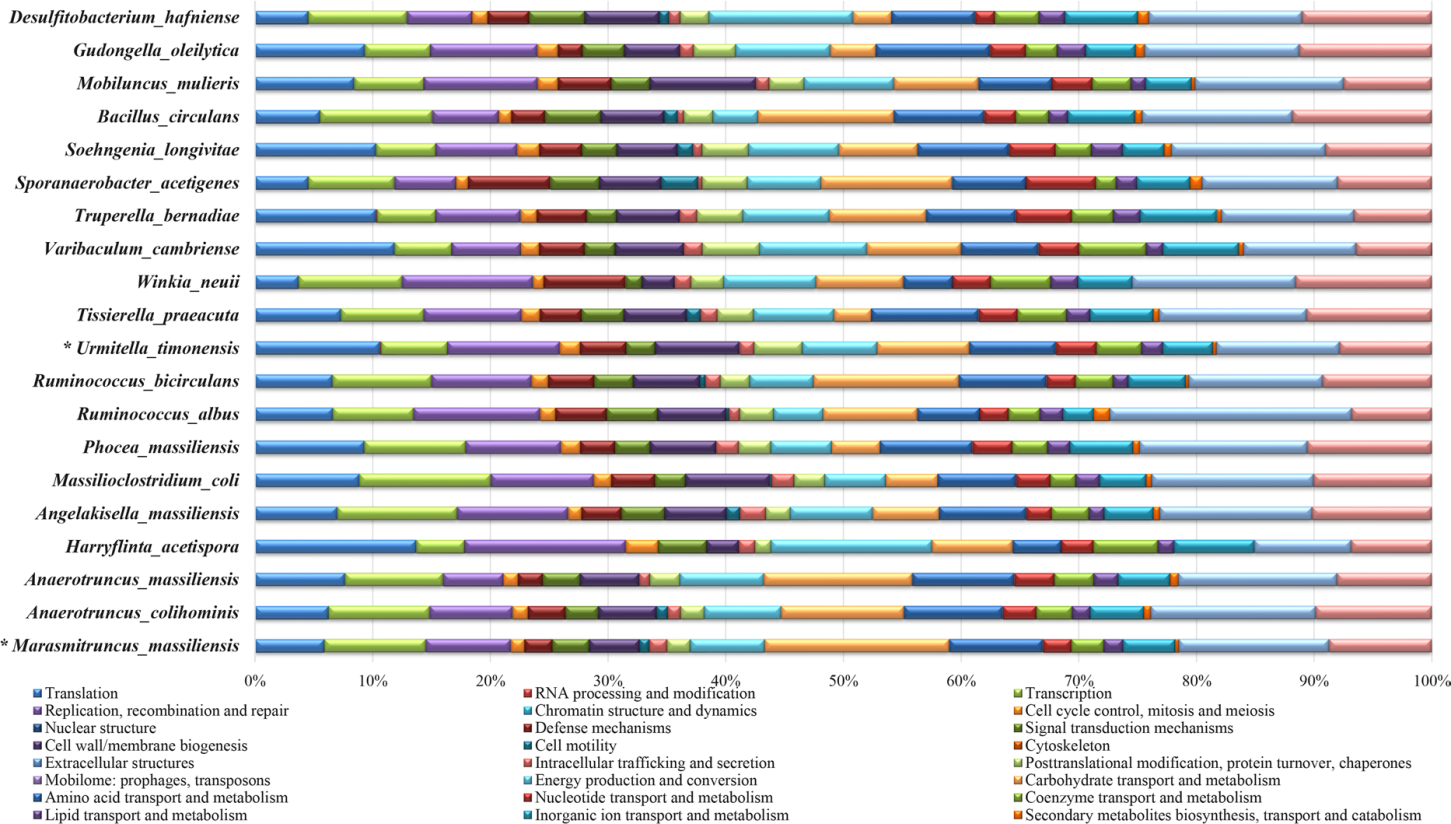
Distribution of functional classes of predicted genes according to the COG of proteins (GOGs) for *Urmitella timonensis* gen. nov., sp. nov. and *Marasmitruncus massiliensis* gen. nov., sp. nov. among closely related bacterial taxa

The genome of strain Marseille-P2918^T^ had a size of 2.13 Mb with a GC content of 50.52% and includes six scaffolds and six contigs, consisting of 1778 predicted coding genes including 21.23% hypothetical proteins. Among these genes, 5.31% are ORFans, 8.1% have a signal peptide according to SignalP-5.0 (Almagro Armenteros et al. 2019) and 25.66% present a transmembrane helix regions according to TMHMM v.2.0 (Krogh et al. 2001). As for the genome of strain Marseille-P3646^T^, it was 3,761,792 bp long consisting of five contigs presenting a 50.04% GC content. The Prokka annotation allowed the prediction of 3594 protein-coding genes. Of those, 12.87% are hypothetical proteins, 4.46% are ORFans, 25.06% have a transmembrane helix region and 8.98% present a signal peptide as presented in Table 2. The distribution of all Prokka annotations, RNA genes (tRNAs, rRNAs), GC content and GC skew is displayed in a graphical circular map for each genome (Figure S3). The distribution of predicted genes into COG functional classes are shown in Fig. 4. For strain Marseille-P2918^T^, 1059 protein-coding genes were assigned to COG categories. No proteins belonging to the chromatin structure and dynamics, nuclear structure, cell motility cytoskeleton, extracellular structures and mobilome COG categories were uncovered. One RNA processing and modification protein was revealed within the genome. For strain Marseille-P3646^T^, 2529 protein-coding genes were assigned to COG categories. No proteins belonging to the RNA processing and modification, chromatin structure and dynamics, nuclear structure, cytoskeleton, extracellular structures and mobilome COG categories were uncovered. Moreover, concerning their metabolic pathways, and according to KEGG BlastKOALA analyses; 58.4% of protein-coding genes (n = 1039) of strain Marseille P2918^T^ are linked to known bacterial metabolic pathways (Table S7) including genetic information processing (27.06%, n = 279), signalling and cellular processing (12.32%, n = 127), carbohydrate metabolism (10.96%, n = 113), amino acid metabolism (6.59% =, n = 68), environmental information processing (5.52%, n = 57). Moreover, strain Marseille P3646^T^ present a similar distribution with 56.1% of protein-coding genes (n = 2018) associated with known metabolic pathways. 21.11% of these genes code for enzymes involved in genetic information processing (n = 426), 15.21% in environmental information processing (n = 307), 14.02% in carbohydrate metabolism (n = 283), 10.3% in signalling and cellular processing (n = 208) and 5.3% in amino acid metabolism (n = 107). This suggests a high growth rate and adaptability to injury for both strains with over 20% of protein-coding genes involved in genetic information processing which could explain their fitness in an altered gut environment such as the gut of malnourished children. In fact, the gut environment of severely malnourished children is an oxidized and nutrient-poor environment (Million et al. 2016).

**Table 2:** Comparison of genomic characteristics

Scaffolds/contigs	*Urmitella timonensis* Marseille-P2918^T^		*Tissierella praeacuta* ATCC 25539^ T^		*Marasmitruncus massiliensis* Marseille-P3646^T^		*Anaerotruncus colihominis* CIP 107754^ T^	
	Value	% of total	Value	% of total	Value	% of total	Value	% of total
Size (bp)	**2,138,714**	**100.00**	**3,206,559**	**100.00**	**3,761,791**	**100.00**	**3,640,133**	**100.00**
G + C content (%)	1,080,460	50.52	971,588	30.3	1,882,513	50.04	1,976,592	54.30
Total genes	**1828**	**100.00**	**3218**	**100.00**	**3653**	**100.00**	**3489**	**100.00**
Protein-coding genes	1778	97.26	3106	96.52	3594	98.38	3359	96.27
RNA genes	65	3.56	73	2.27	44	1.20	59	1.69
Hypothetical proteins	388	21.23	ND	ND	470	12.87	ND	ND
Proteins with peptide signals	144	8.10	ND	ND	328	8.98	ND	ND
Number of proteins associated to ORFans	97	5.31	ND	ND	163	4.46	ND	ND
Genes with transmembrane helices	469	25.66	ND	ND	916	25.06	ND	ND
Genes associated with PKS or NRPS	0	0.00	ND	ND	0	0.00	ND	ND
No. of antibiotic resistance genes	1	0.05	ND	ND	0	0.00	ND	ND

the bold value refers to the total (100%) value

Antibiotic resistance screening only allowed the detection of tet(O) in strain Marseille-P2918^T^. This gene confers resistance to doxycycline, tetracycline and minocycline. In addition, for the prediction of new antibiotic resistance genes, we noticed the presence of a coding gene, annotated as a hypothetical protein according to Prokka, presenting a functional domain that confers resistance to nitroimidazole in strain Marseille-P3646^T^. This gene has been detected by 53.42% similarity and 98.77% coverage with NimE. The prediction of its function by 3D structure, BLAST CDD (Conserved domain database), and Motif search showed that this hypothetical protein has a Pyridoxamine 5'-phosphate oxidase activity, which is known as a function of Nim genes according to Uniprot. Thus, strain Marseille-P3646^T^ has a potential new Nitroimidazole resistance gene, present the functional domain necessary for this resistance.

**Fig. 5:**
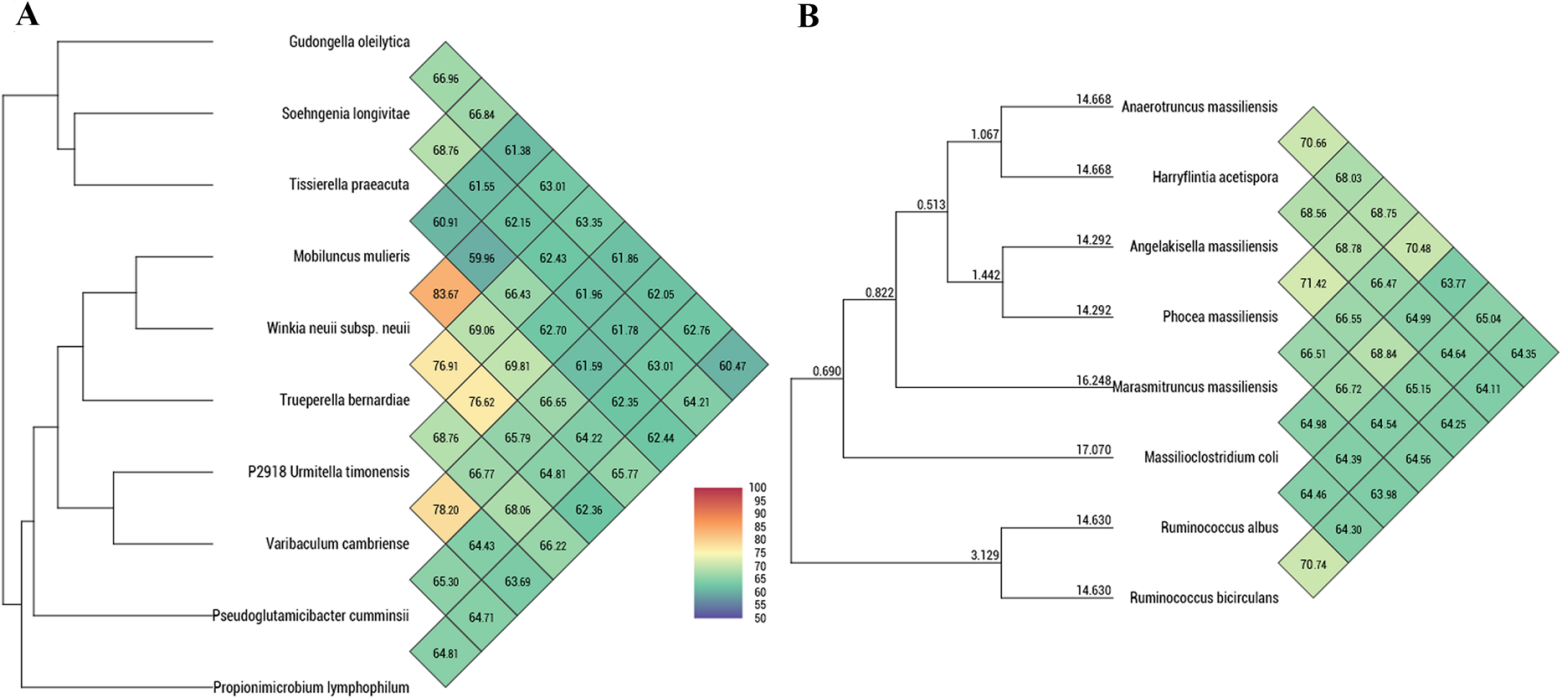
Heatmap generated with OrthoANI values calculated using the OAT software between *Urmitella timonensis* (**A**) and *Marasmitruncus massiliensis* (**B**), with their respective closely related taxa with standing in nomenclature

Genomic characteristics of our strains were compared to those of closely related species with an available genome. OrthoANI values among closely related species to strain Marseille-P2918^T^ ranged from 61.86% between strain Marseille-P2918^T^ and *Gudongella oleilytica;* and 78.20% between strain Marseille-P2918^T^ and *Varibaculum cambriense* (Fig. 5A). When strain Marseille-P3646^T^ was compared to closely related species, values ranged from 63.98% with *Ruminococcus bicirculans* to 70.48% with *Anaerotruncus massiliensis* (Fig. 5B).

Furthermore, dDDH values (Tables S8 and S9) are all under 70%, with the highest values of 25.5% between *Urmitella timonensis* strain Marseille-P2918^T^ and *Desulfitobacterium hafniense*, and 34.5% between *Marasmitruncus massiliensis* strain Marseille-P3646^T^ and *Massilioclostridium coli.* Moreover, the analyses of their amino acid sequences showed the maximum %AAI for Marseille-P3646^T^ with *Clostridiales bacterium* (60%) and for Marseille-P2918^T^ with *Varibaculum cambriense* (86%)*.* This confirms that all the studied strains are distinct, previously unknown taxa.

## Conclusion

In this study, the culturomics approach enabled us to isolate two novel isolates, described here using the taxono-genomics based on their main phenotypic and genomic characteristics. According to their 16S rRNA gene and genome sequence divergence, and according to the threshold proposed by Stackbrandt and Ebers for defining new species, we propose *Urmitella timonensis* gen. nov., sp. nov., with strain Marseille-P2918 as its type strain, and *Marasmitruncus massiliensis’* gen. nov., sp. nov.*,* with strain Marseille-P3646 as its type strain, both of which have been isolated from the faeces of a child suffering from marasmus, a form of severe acute malnutrition.

### Description of *Urmitella* gen. nov.

(Ur.mit.tel’la N.L. Gen.fem, to refer to URMITE, the name of the laboratory where the strain was isolated, Marseille, France)*.*

*Urmitella* gen. nov. is classified within the family *Tissierellaceae*, order *Tissierellales*, class *Tissierellia*, phylum *Firmicutes* as the type strain Marseille-P2918 exhibits a 6.29% 16S rRNA gene divergence with *Tissierella preacuta* strain NCTC 11158^T^. Cells are Gram-stain negative, motile and spore forming bacilli. Strictly anaerobic. Oxidase and catalase negative.

The type species, *Urmitella timonensis*, was isolated from the gut of a child suffering from marasmus.

### Description of *Urmitella timonensis* sp. nov.

(ti.mo.nen’sis L. adj. fem. to refer to the Timone, the name of the main hospital of Marseille, France, where the strain was isolated).

Cells of strain Marseille-P2918^T^ are Gram-stain negative bacilli. Cells size were 5.33 ± 1.5 µm in length and 0.70 ± 0.09 µm in width. No oxidase and catalase activities were found. *Urmitella timonensis* is motile and spore forming. Colonies are circular, smooth, very small and pale grey, with a diameter of 0.3–1 mm on blood agar. Strictly anaerobic. Optimum growth occurs at 37 °C in an anaerobic atmosphere. The major fatty acid is hexadecanoic acid (40%). The habitat is the human gut. The genome of strain Marseille-P2918^T^ is 2.14 Mbp long with 50.52% of GC content. The 16S rRNA and genome sequences are available in the EMBL-EBI database under accession numbers LT598554 and FQLW01000000, respectively. The type strain Marseille-P2918^T^ (= CSUR P2918 = DSM103634) was isolated from the stool sample from a Senegalese girl suffering from marasmus.

### Description of *Marasmitruncus* gen. nov.

*Marasmitruncus* (Ma.ras.mi.trun’cus. N.L. masc. n. *marasmus*, from Gr. masc. n. *marasmos*, a causing to die away; N.L. masc. n. *truncus*, stick; N.L. masc. n. *Marasmitruncus*, a rod isolated from a patient with marasmus).

*Marasmitruncus* gen. nov. is classified within the family *Oscillospiraceae*, order *Eubacteriales*, class *Clostridia* and phylum *Firmicutes* as its type strain, Marseille-P3646, has a 93.11% with its closest relative, *Anaerotruncus colihominis* strain 14565^ T^. Bacterial cells are strictly anaerobic, Gram-stain negative, rod-shaped, and motile with negative catalase and oxidase activities.

### Description of *Marasmitruncus massiliensis* gen. nov., sp. nov.

Description of *Marasmitruncus massiliensis* gen. nov., sp. nov. (mas.si.li.en’sis L. masc. adj., *massiliensis* pertaining to Massilia, the Roman name of the city of Marseille, where this bacterium was discovered).

Cells are Gram-stain negative bacilli. Cells sizes were 2.71 ± 0.6 µm in length and 0.42 ± 0.06 µm in diameter. No oxidase and catalase activities were found. Marseille-P3646^T^ is motile and spore forming. Colonies are circular, smooth, very small with a diameter of 0.5–2 mm on blood agar. Optimum growth occurs at 37 °C in an anaerobic and microaerophilic atmosphere. The major fatty acid is 12-methyl-tetradecanoic acid.

The genome of strain Marseille-P3646^T^ is 3,761,792 bp long with 50.04% of G + C content. The 16S rRNA and genome sequences are available in the EMBL-EBI database under accession numbers LT725660.1and FYDE00000000, respectively. The habitat is the human gut. The type strain Marseille-P3646^T^ (= CSUR P3646 = CCUG72353) was isolated from the stool sample from a Senegalese girl suffering from marasmus.

### Strain deposition

Strains Marseille-P2918^T^ and Marseille-P3646^T^ were both deposited in the Collection des Souches de l’Unité des Rickettsies (CSUR) under deposition numbers CSUR P2918 and CSUR P3646 respectively. Additionally, strain Marseille-P2918^T^ was deposited in the Deutsche Sammlung von Mikroorganismen und Zellkulturen (DSMZ) under number DSM 103634 whereas strain Marseille-P3646^T^ was deposited in the Culture Collection University Of Gothenburg (CCUG) under number CCUG 72353.

## Supplementary Information

Below is the link to the electronic supplementary material.

Supplementary file1 (DOCX 6466 kb)
