# Supplementary material for: Draft genomes and descriptions of Urmitella timonensis gen. nov., sp. nov. and Marasmitruncus massiliensis gen. nov., sp. nov., isolated from severely malnourished African children using culturomics
Source: Antonie Van Leeuwenhoek. 2022 Sep 23;115(11):1349–61. doi: 10.1007/s10482-022-01777-x (PMC9584879; doi:10.1007/s10482-022-01777-x)

**Draft Genomes and descriptions of *Urmitella timonensis* gen. nov., sp. nov. and *Marasmitruncus massiliensis* gen. nov., sp. nov*.,* two new genera isolated from severely malnourished African children using culturomics**

Sara Bellali^1†^, Gabriel Haddad^1,2†^, Thi-Phuong-Thao Pham^1^, Rim Iwaza^1,2^, Ahmad Ibrahim^1,2^, Nicholas Armstrong^1^, Amael Fadlane^1^, Carine Couderc^1^, Aldiouma Diallo^3^, Cheikh Sokhna^3,4^, Matthieu Million^1^, Didier Raoult^1,2^ and Maryam Tidjani Alou^1*^

^1^Aix Marseille Université, MEPHI, URMITE, UM63, CNRS 7278, IRD 198, INSERM 1095, ^2^IHU Méditerranée Infection, 19-21 Boulevard Jean Moulin, 13385 Marseille Cedex 05

^3^Campus Commun UCAD-IRD of Hann, Dakar, Senegal

^4^Aix Marseille Univ, IRD, AP-HM, SSA, VITROME, Marseille, France

^†^ These authors contributed equally to this work

^*^**Corresponding author**: Maryam Tidjani Alou, **E-mail**: tidjani_maryam@hotmail.com

**Table S1:** *Urmitella timonensis* gen.nov., sp. nov. *and Marasmitruncus massiliensis* gen. nov., sp. nov. reactions result with API® ZYM. (±) weakly positive reaction; (−) negative reaction (+), positive reaction.

| **Active component** | ***Urmitella timonensis*** | ***Marasmitruncus massiliensis*** |
| --- | --- | --- |
| Alkaline phosphatase | + | - |
| Esterase (C4) | - | + |
| Esterase lipase (C8) | - | ± |
| Lipase (C14) | - | - |
| Leucine arylamidase | + | - |
| Valine arylamidase | - | - |
| Cystine arylamidase | - | - |
| Trypsin | - | - |
| α-chymotrypsin | - | - |
| Acid phosphatase | + | + |
| Naphthol-AS-BI-phosphohydrolase | + | + |
| α-galactosidase | - | - |
| β-galactosidase | - | - |
| β-glucuronidase | - | - |
| α-glucosidase | + | - |
| β-glucosidase | - | - |
| N-acetyl-β-glucosaminidase | - | - |
| α-mannosidase | - | - |
| α-fucosidase | - | - |

**Table S2:** *Urmitella timonensis* gen. nov., sp. nov. and *Marasmitruncus massiliensis* gen. nov., sp. nov. reaction results with API® 20A. (±) weakly positive reaction; (−) negative reaction (+), positive reaction.

| **Active component** | ***Urmitella timonensis*** | ***Marasmitruncus massiliensis*** |
| --- | --- | --- |
| L-tryptophane | **-** | **-** |
| Urea | **-** | **-** |
| D-glucose | **+** | **±** |
| D-mannitol | **+** | **-** |
| D-lactose | **+** | **-** |
| D-saccharose | **+** | **-** |
| D-maltose | **+** | **-** |
| Salicin | **+** | **-** |
| D-xylose | **-** | **±** |
| L-arabinose | **+** | **-** |
| Gelatin | **+** | **-** |
| Esculin ferric citrate | **-** | **-** |
| Glycerol | **+** | **-** |
| D-cellobiose | **+** | **-** |
| D-mannose | **-** | **-** |
| D-melezitose | **+** | **-** |
| D-raffinose | **-** | **-** |
| D-sorbitol | **+** | **-** |
| L-rhamnose | **+** | **-** |
| D-trehalose | **-** | **-** |

**Table S3:** *Urmitella timonensis* gen.nov., sp. nov. *and Marasmitruncus massiliensis* gen.nov., sp. nov*.* reactions result with API® 50CH. (±) weakly positive reaction; (−) negative reaction (+), positive reaction.

| **Enzyme assayed for** | ***Urmitella timonensis*** | ***Marasmitruncus massiliensis*** |
| --- | --- | --- |
| control | - | - |
| Glycerol | + | - |
| Erythritol | + | - |
| D-arabinose | + | - |
| L-arabinose | - | - |
| D-ribose | + | ± |
| D-xylose | + | - |
| L-xylose | - | - |
| D-adonitol | + | ± |
| Methyl-βD-xylopyranoside | - | ± |
| D-galactose | - | - |
| D-glucose | - | - |
| D-fructose | - | ± |
| D-mannose | + | ± |
| L-sorbose | + | ± |
| L-rhamnose | + | ± |
| Dulcitol | + | ± |
| Inositol | - | ± |
| D-mannitol | - | ± |
| D-sorbitol | + | ± |
| Methyl-αD-mannopyranoside | + | - |
| Methyl-αD-glucopyranoside | + | - |
| N-acetylglucosamine | + | - |
| Amygdalin | + | ± |
| Arbutin | + | - |
| Esculin ferric citrate | + | - |
| Salicin | + | ± |
| D-cellobiose | + | - |
| D-maltose | + | ± |
| D-lactose | + | ± |
| D-melibiose | - | ± |
| D-saccharose | + | - |
| D-trehalose | + | ± |
| Inulin | + | + |
| D-melezitose | + | - |
| D-raffinose | + | - |
| Amidon | + | - |
| Glycogen | + | - |
| Xylitol | + | - |
| Gentiobiose | + | - |
| D-turanose | + | ± |
| D-lyxose | + | - |
| D-tagatose | + | ± |
| D-fucose | + | - |
| L-fucose | - | ± |
| D-arabitol | - | - |
| L-arabitol | + | ± |
| Potassium gluconate | - | ± |
| Potassium 2-ketogluconate | - | - |
| Potassium 5-ketogluconate | + | + |

**Table S4:** Cellular fatty acid composition (in %). Mean peak area percentage ± standard deviation; TR= trace amounts < 1 %

| **Fatty acids** | **IUPAC name** | ***Urmitella timonensis***  Marseille-P2918^T^ | ***Marasmitruncus massiliensis***  Marseille-P2918^T^ |
| --- | --- | --- | --- |
| **13:0** | **Tridecanoic acid** | TR | 1.0 ± 0.1 |
| **13:0 anteiso** | **10-methyl-Dodecanoic acid** | - | - |
| **14:0** | **Tetradecanoic acid** | 6.1 ± 0.5 | 1.0 ± 0.1 |
| **14:0 iso** | **12-methyl-Tridecanoic acid** | - | 5.2 ± 0.1 |
| **15:0** | **Pentadecanoic acid** | 2.8 ± 0.2 | TR |
| **15:0 iso** | **13-methyl-tetradecanoic acid** | TR | 23.1 ± 0.3 |
| **15:0 anteiso** | **12-methyl-tetradecanoic acid** | TR | 38.6 ± 2.4 |
| **15:1** | **Pentadecenoic acid** | TR | - |
| **16:0** | **Hexadecanoic acid** | 39.7 ± 3.1 | 15.4 ± 0.4 |
| **16:0 9,10-methylene** | **2-hexyl-Cyclopropaneoctanoic acid** | TR | - |
| **16:0 iso** | **14-methyl-Pentadecanoic acid** | - | 1.8 ± 0.1 |
| **16:1n7** | **9-Hexadecenoic acid** | TR | TR |
| **16:1n9** | **7-Hexadecenoic acid** | - | - |
| **17:0** | **Heptadecanoic acid** | TR | TR |
| **17:0 iso** | **15-methyl-Hexadecanoic acid** | - | 1.8 ± 2.1 |
| **17:0 anteiso** | **14-methyl-Hexadecanoic acid** | TR | 2.4 ± 0.1 |
| **18:0** | **Octadecanoic acid** | 9.1 ± 1.8 | 1.5 ± 0.1 |
| **18:0 9,10-methylene** | **2-octyl-Cyclopropaneoctanoic acid** | 1.0 ± 0.1 | - |
| **18:1n7** | **11-Octadecenoic acid** | 4.7 ± 0.1 | TR |
| **18:1n9** | **9-Octadecenoic acid** | 25.4 ± 2.5 | 2.4 ± 0.1 |
| **18:2n6** | **9,12-Octadecadienoic acid** | 8.5 ± 1.5 | 1.3 ± 0.1 |

**Table S5:** Antibiotic resistance tests. values are in µg/mL.

|  | ***Urmitella timonensis***  Marseille-P2918^T^ | ***Marasmitruncus massiliensis***  Marseille-P2918^T^ |
| --- | --- | --- |
| **Benzylpenicillin** | 0.32 | 0.032 |
| **Oxacillin** | 0.5 | 0.75 |
| **Ceftazidime** | 0.5 | <0.016 |
| **Tobramycin** | 0.64 | >256 |
| **Amikacin** | >256 | 16 |
| **Amoxicillin** | 0.125 | <0.016 |
| **Ceftriaxone** | 0.64 | <0.016 |
| **Imipenem** | 0.125 | 0.25 |
| **Vancomycin** | 3 | 1.5 |
| **Doxycycline** | <0.016 | <0.016 |
| **Clindamycin** | <0.016 | <0.016 |
| **Trimethoprim/sulfamethoxazole** | >32 | >32 |
| **Ciprofloxacin** | 0.94 | >32 |
| **Rifampicin** | 0.006 | 0.016 |
| **Linezolid** | 1.5 | <0.016 |
| **Colistin** | >256 | >256 |

**Table S6: 16S sequence similarity between strains Marseille-P2918^T^ and Marseille-P3646^T^ and type species from the Tissierellaceae and Oscillospiraceae families respectively.**

|  | Strain Marseille-P2918^T^ (LT598554) | Strain Marseille-P3646^T^ (LT725660) |
| --- | --- | --- |
| *Tissierella praeacuta* NCTC 11158^T^ (X80832) | 93.71% |  |
| *Gudongella oleilytica* DSM 28124^T^  (KP119717) | 93.33% |  |
| *Soehngenia saccharolytica* DSM 12858^T^ (GQ461828) | 91.73% |  |
| *Sporanaerobacter acetigenes* DSM 13106T (AF358114) | 91.13% |  |
| *Keratinibaculum paraultunense* DSM 26752^T^ (KC188660) | 90.92% |  |
| *Schnuerera ultunensis* ATCC 700254^T^(Z69293) | 90.38% |  |
| *Tepidimicrobium ferriphilum* DSM 16624^T^ (GQ461826) | 89.60% |  |
| *Anaerosalibacter bizertensis* DSM 23801^T^ (HQ534365) | 89.57% |  |
| *Anaerotruncus colihominis* strain 14565^T^ (AJ315980) |  | 93.11% |
| *Acetanaerobacterium elongatum* JCM 12359^T^ (AY487928) |  | 92.33% |
| *Harryflintia acetispora* CECT 8892^T^ (KU999999) |  | 92.21% |
| *Tepidibaculum saccharolyticum* DSM 28577^T^ (MH200617) |  | 92.13% |
| *Hydrogenoanaerobacterium saccharovorans* DSM 24774^T^ (EU158190) |  | 91.18% |
| *Ruminococcus flavefaciens* ATCC 19208^a^(KP689131) |  | 87.71% |

Table S7: Metabolic pathway categories for *Urmitella timonensis* gen. nov., sp. nov. and *Marasmitruncus massiliensis* gen. nov., sp. nov.

| **Scaffolds/ contigs** | ***Urmitella timonensis***  **Marseille-P2918^T^** | | ***Marasmitruncus massiliensis***  **Marseille-P3646^T^** | |
| --- | --- | --- | --- | --- |
|  | **Value** | **% of total** | **Value** | **% of total** |
| **Genetic information processing** | 305 | 29.36 | 471 | 23.03 |
| **Signaling and cellular processing** | 175 | 16.84 | 354 | 17.31 |
| **Carbohydrate metabolism** | 113 | 10.88 | 283 | 13.84 |
| **Amino acid metabolism** | 79 | 7.60 | 119 | 5.82 |
| **Environmental information processing** | 59 | 5.68 | 310 | 15.16 |
| **Metabolism of cofactors and vitamins** | 54 | 5.20 | 69 | 3.37 |
| **Unclassified metabolism** | 87 | 8.37 | 190 | 9.29 |
| **Nucleotide metabolism** | 43 | 4.14 | 64 | 3.13 |
| **Energy metabolism** | 36 | 3.46 | 52 | 2.54 |
| **Other proteins metabolism** | 32 | 3.08 | 43 | 2.10 |
| **Lipid metabolism** | 22 | 2.12 | 42 | 2.05 |
| **Glycan biosynthesis and metabolism** | 16 | 1.54 | 20 | 0.98 |
| **Metabolism of terpenoids and polyketides** | 8 | 0.77 | 7 | 0.34 |
| **Organismal Systems** | 5 | 0.48 | 5 | 0.24 |
| **Human diseases** | 3 | 0.29 | 12 | 0.59 |
| **Xenobiotics biodegradation and metabolism** | 2 | 0.19 | 4 | 0.20 |
| **Total** | **1,039** | **100.00** | **2,045** | **100.00** |

**Table S8:** digital DDH values obtained by comparison of all studied genomes compared to *Urmitella timonensis*.

| **Species** | ***T. praeacuta*** | ***S. acetigenes*** | ***S. longivitae*** | ***U. timonensis*** | ***K. paraultunense*** | ***G. oleilytica*** | ***D. hafniense*** | ***B. circulans*** |
| --- | --- | --- | --- | --- | --- | --- | --- | --- |
| ***Tissierella praeacuta*** | **100** | **25.3**  **[23.0-27.8]** | **21**  **[18.7-23.4]** | **19**  **[16.8-21.3]** | **18.7**  **[16.5-21.1]** | **23**  **[20.7-25.5]** | **29.5**  **[27.1-32.0]** | **23.2**  **[20.9-25.7]** |
| ***Sporanaerobacter acetigenes*** |  | **100** | **23.3**  **[21.0-25.8]** | **3.7**  **[2.8-4.8]** | **15.2**  **[13.2-17.5]** | **24.8**  **[22.5-27.3]** | **3.7**  **[2.8-4.8]** | **3.7**  **[2.8-4.8]** |
| ***Soehngenia longivitae*** |  |  | **100** | **18.4**  **[16.3-20.8]** | **18.2**  **[16.1-20.6]** | **21.8**  **[19.6-24.3]** | **36.6**  **[34.2-39.2]** | **22**  **[19.7-24.4]** |
| ***Urmitella timonensis*** |  |  |  | **100** | **18.4**  **[16.2-20.7]** | **18.1**  **[15.9-20.4]** | **25.5**  **[23.2-28.0]** | **19.9**  **[17.7-22.3]** |
| ***Keratinibaculum paraultunense*** |  |  |  |  | **100** | **22.4**  **[20.1-24.8]** | **30.8**  **[28.4-33.3]** | **22.9**  **[20.7-25.4]** |
| ***Gudongella oleilytica*** |  |  |  |  |  | **100** | **30.7**  **[28.3-33.2]** | **22.7**  **[20.4-25.1]** |
| ***Desulfitobacterium hafniense*** |  |  |  |  |  |  | **100** | **35.8**  **[33.3-38.3]** |
| ***Bacillus circulans*** |  |  |  |  |  |  |  | **100** |

**Table S9:** digital DDH values obtained by comparison of all studied genomes compared to *Marasmitruncus massiliensis*.

| **Species** | ***A. massiliensis*** | ***A. massiliensis*** | ***H. acetispora*** | ***M. massiliensis*** | ***M. coli*** | ***P. massiliensis*** | ***R. albus*** | ***R. bicirculans*** |
| --- | --- | --- | --- | --- | --- | --- | --- | --- |
| ***Anaerotruncus massiliensis*** | **100** | **27.6**  **[25.3–30.1]** | **19.1**  **[16.9–21.5]** | **17.8**  **[15.7–20.2]** | **34.7**  **[32.3–37.2]** | **21.7**  **[19.4–24.1]** | **30.3**  **[28.0–32.9]** | **32.8**  **[30.4–35.3]** |
| ***Angelakisella massiliensis*** |  | **100** | **26.7**  **[24.4–29.2]** | **23.1**  **[20.8–25.6]** | **43.9**  **[41.3–46.4]** | **29.8**  **[27.4–32.3]** | **25.9**  **[23.6–28.4]** | **30.6**  **[28.2–33.1]** |
| ***Harryflintia acetispora*** |  |  | **100** | **19.7**  **[17.5–22.1]** | **49.5**  **[46.9–52.1]** | **22.9**  **[20.6–25.3]** | **24.8**  **[22.5–27.3]** | **29.2**  **[26.9–31.7]** |
| ***Marasmitruncus massiliensis*** |  |  |  | **100** | **34.5**  **[32.1–37.0]** | **20.1**  **[17.8–22.5]** | **30.8**  **[28.4–33.3]** | **31.5**  **[29.1–34.0]** |
| ***Massilioclostridium coli*** |  |  |  |  | **100** | **38.2**  **[35.8–40.7]** | **28**  **[25.7–30.5]** | **28.7**  **[26.3–31.2]** |
| ***Phocea massiliensis*** |  |  |  |  |  | **100** | **28.3**  **[26.0–30.8]** | **32.8**  **[30.4–35.3]** |
| ***Ruminococcus albus*** |  |  |  |  |  |  | **100** | **24.9**  **[22.5–27.3]** |
| ***Ruminococcus bicirculans*** |  |  |  |  |  |  |  | **100** |

**Figure S1.** MALDI-TOF MS reference spectrum of *Urmitella timonensis* gen. nov., sp. nov. and *Marasmitruncus massiliensis* gen. nov., sp. nov. The reference spectrum was generated by comparison of spectra from 12 individual colonies.


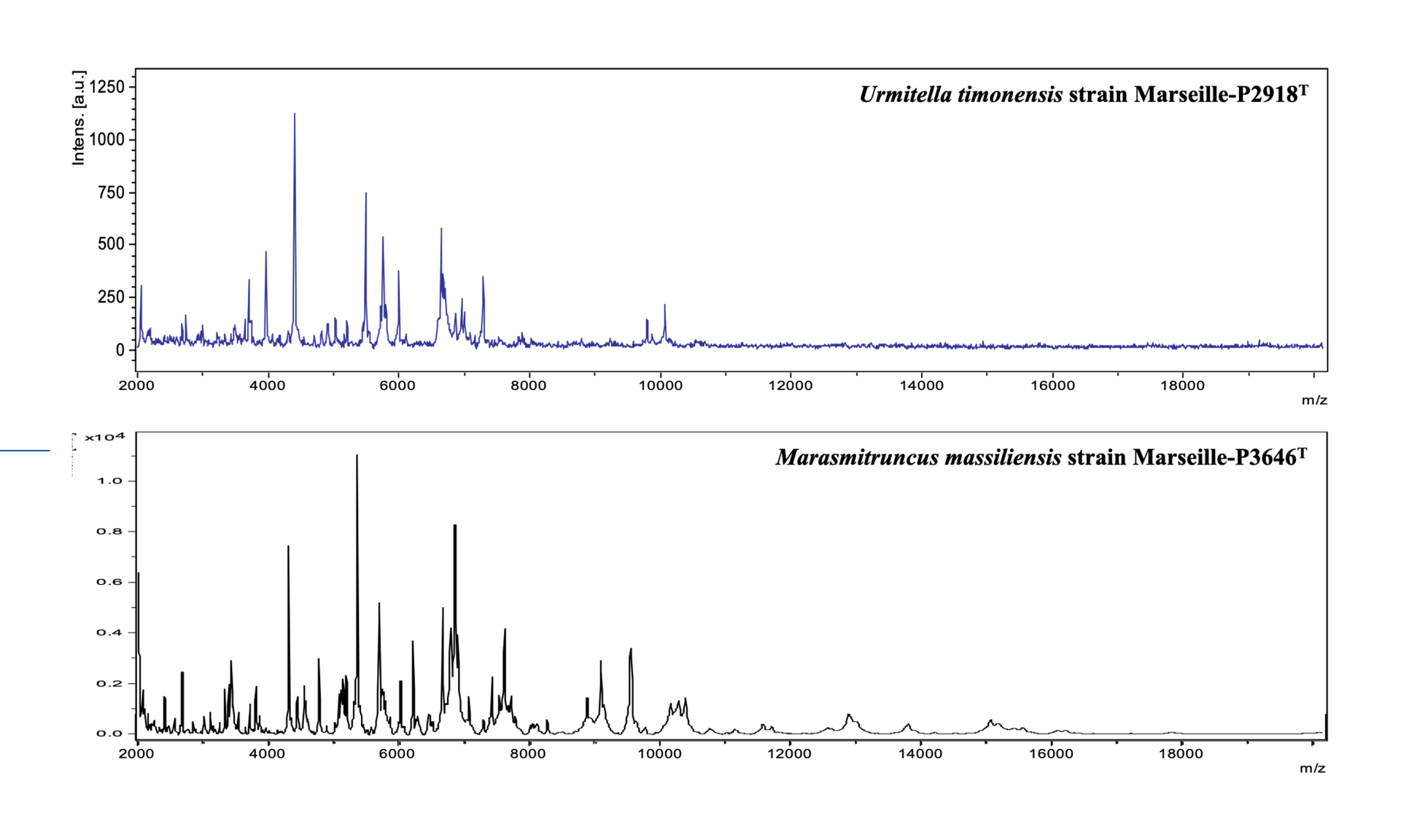


**Figure S2.** Maximum Parsimony phylogenetic analysis of strains Marseille-P2918^T^ and Marseille-P3646^T^. The evolutionary history was inferred using the Maximum Parsimony method. Tree #1 out of 2 most parsimonious trees (length = 985) is shown. The consistency index is ( 0.444181), the retention index is ( 0.471783), and the composite index is 0.247626 ( 0.209557) for all sites and parsimony-informative sites (in parentheses). The percentage of replicate trees in which the associated taxa clustered together in the bootstrap test (1000 replicates) are shown next to the branches [1]. The MP tree was obtained using the Subtree-Pruning-Regrafting (SPR) algorithm (pg. 126 in ref. [2]) with search level 1 in which the initial trees were obtained by the random addition of sequences (10 replicates). The tree is drawn to scale , with branch lengths calculated using the average pathway method [see pg. 132 in ref. 2] and are in the units of the number of changes over the whole sequence . The analysis involved 17 nucleotide sequences. Codon positions included were 1st+2nd+3rd+Noncoding. All positions containing gaps and missing data were eliminated. There were a total of 1335 positions in the final dataset. Evolutionary analyses were conducted in MEGA7 [3].


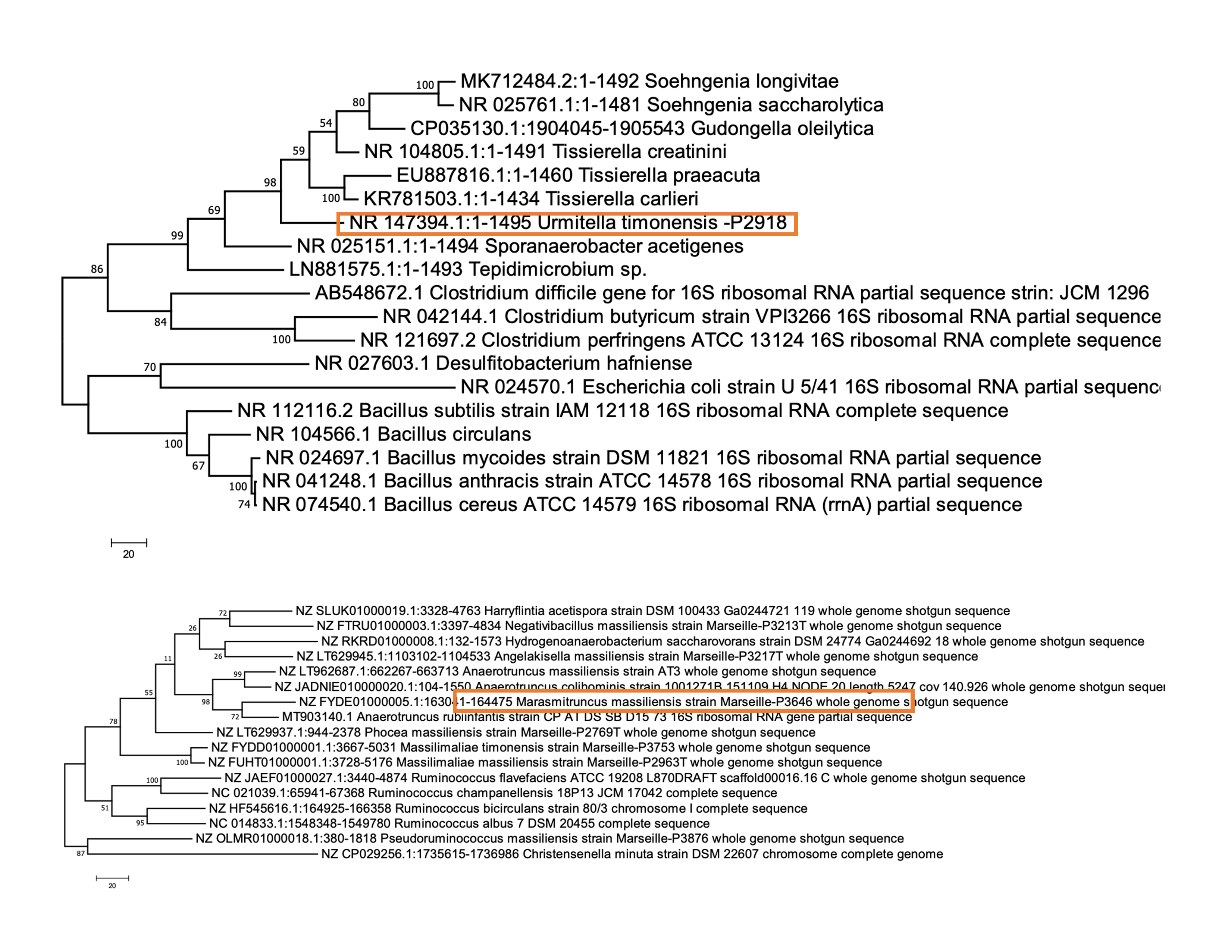


**Figure S3.** Circular genome of *Urmitella timonensis* gen. nov., sp. nov. (**A**) and *Marasmitruncus massiliensis* gen. nov., sp. nov. (**B**) displaying – from inside – the Open Reading Frames, GC content, GC skew, contigs and Prokka annotation. Map was built using CGview.


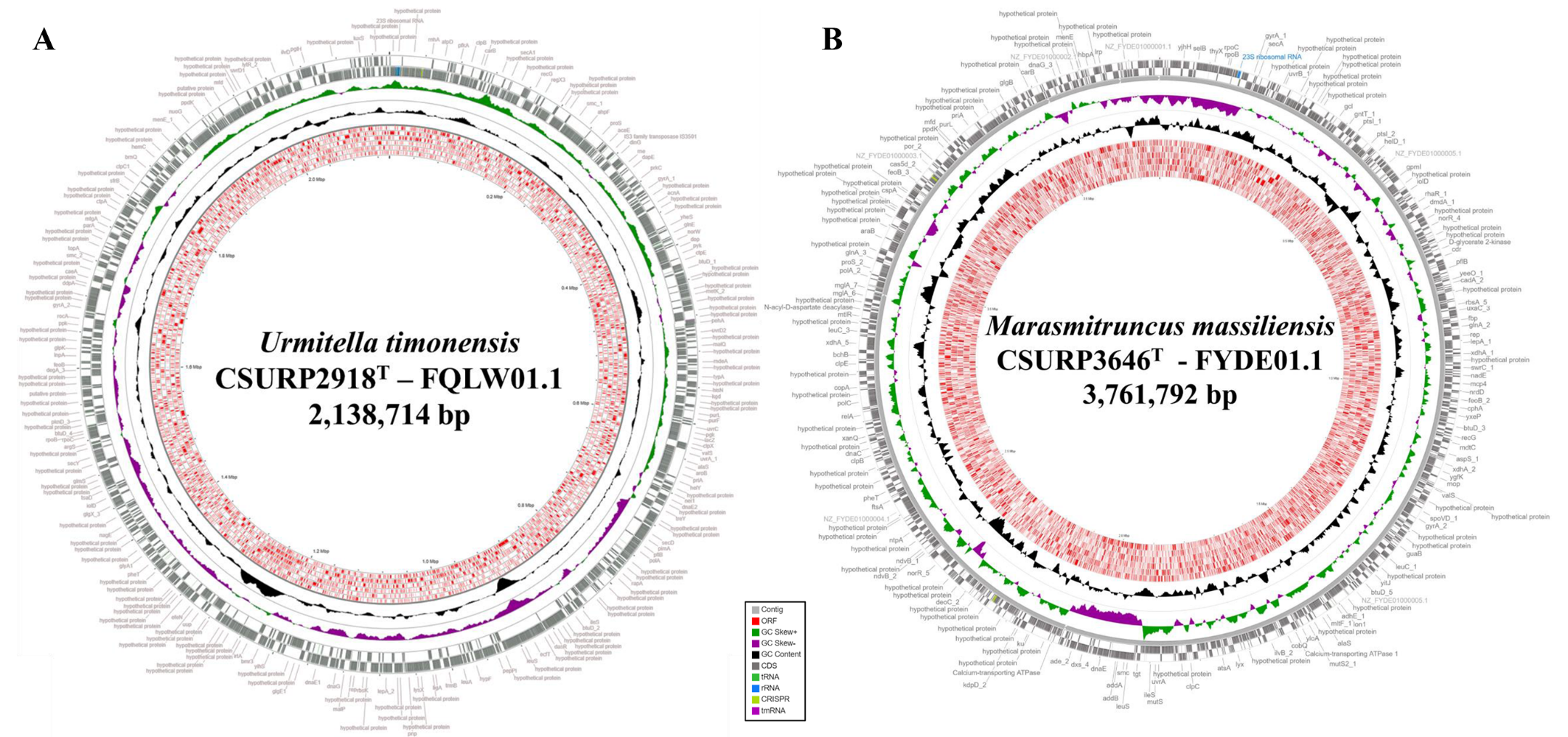

Supplement: Supplementary file 1 — Supplementary file1 (DOCX 6466 kb) [file 10482_2022_1777_MOESM1_ESM.docx]
